# The unique characteristics of COVID-19 coagulopathy

**DOI:** 10.1186/s13054-020-03077-0

**Published:** 2020-06-18

**Authors:** Toshiaki Iba, Jerrold H. Levy, Jean Marie Connors, Theodore E. Warkentin, Jecko Thachil, Marcel Levi

**Affiliations:** 1 Department of Emergency and Disaster Medicine, Juntendo University Graduate School of Medicine, 2-1-1 Hongo Bunkyo-ku, Tokyo, 113-8421 Japan; 2 Department of Anesthesiology, Critical Care, and Surgery, Duke University School of Medicine, Durham, NC USA; 3 Hematology Division Brigham and Women’s Hospital, Harvard Medical School, Boston, MA USA; 4 Department of Pathology and Molecular Medicine, and Department of Medicine, McMaster University, Hamilton, Canada; 5 Department of Haematology, Manchester Royal Infirmary, Manchester, UK; 6 Department of Medicine, University College London Hospitals NHS Foundation Trust and Cardio-metabolic Programme-NIHR UCLH/UCL BRC London, London, UK

**Keywords:** COVID-19, Coagulopathy, Disseminated intravascular coagulation, Hemophagocytic syndrome, Antiphospholipid syndrome, Thrombotic microangiopathy

## Abstract

Thrombotic complications and coagulopathy frequently occur in COVID-19. However, the characteristics of COVID-19-associated coagulopathy (CAC) are distinct from those seen with bacterial sepsis-induced coagulopathy (SIC) and disseminated intravascular coagulation (DIC), with CAC usually showing increased D-dimer and fibrinogen levels but initially minimal abnormalities in prothrombin time and platelet count. Venous thromboembolism and arterial thrombosis are more frequent in CAC compared to SIC/DIC. Clinical and laboratory features of CAC overlap somewhat with a hemophagocytic syndrome, antiphospholipid syndrome, and thrombotic microangiopathy. We summarize the key characteristics of representative coagulopathies, discussing similarities and differences so as to define the unique character of CAC.

## Background

The high mortality and its relationship with thromboembolic diseases in COVID-19 have increasingly attracted attention [1, 2]. D-dimer has been repeatedly reported to be a useful biomarker associated with the severity of disease [3] and is a predictor of adverse outcomes [4]. The high incidence of venous thromboembolism (VTE) and the importance of giving anticoagulant thromboprophylaxis is stated in guidance documents [5] and supported by consecutive autopsy findings noting frequent deep vein thrombosis in 7 of 12 COVID-19 patients (58%) with complicating pulmonary embolism in 4 patients (33%) [6]. An increased incidence of arterial thromboses such as stroke and acute coronary syndromes has also been reported in COVID-19 [7]. The effectiveness of prophylactic and therapeutic anticoagulant use in this context is controversial. The initial Wuhan description reported a lower mortality heparin-treated patients with D-dimer levels over 3.0 μg/mL (32.8% vs 52.4% not treated, *P* = 0.017) or sepsis-induced coagulopathy (SIC) (40.0% vs 64.2% not treated, *P* = 0.029) [8]. In another study, Paranjpe et al. [9] analyzed 2773 COVID-19 patients of whom only 28% received anticoagulant therapy; in patients requiring mechanical ventilation (*n* = 395), in-hospital mortality was lower in patients who received systemic anticoagulation at 29.1% with a median survival of 21 days compared to 62.7% mortality and median survival of 9 days in patients not treated with anticoagulation. Despite the retrospective data, the report emphasizes the role of hypercoagulability in COVID-19 and the role of anticoagulation. However, the pathophysiology of COVID-19-associated coagulopathy (CAC) is complex and likely to differ in important ways from the standard mechanisms of thrombosis reported in critically ill patients. This review will compare and contrast various well-characterized types of coagulopathy with CAC (Table 1).

**Table 1:** The similarities and the differences in thrombosis and laboratory data between COVID-19 and differential diseases

	Primary cause and target of coagulopathy	Thrombo-embolism	Platelet count	D-dimer	PT/aPTT	fibrinogen	Anti-thrombin	Activated complement system/VWF	Antiphospho-lipid antibody	Inflammatory cytokines (IL-1β, IL-6)
COVID-19	Macrophage/endothelial cell	Microthrombosis/venous thrombosis	↑〜↓	↑	→〜↑	↑	→	+	+	↑
DIC/SIC	Macrophage/endothelial cell	Microthrombosis	↓	↑	↑	→〜↓	↓	–	–	↑
HPS	Inflammatory cytokines	Microthrombosis/venous thrombosis	↓	→	→	→	→	–	–	↑
APS	Antiphospho-lipid antibody	Arterial/venous thrombosis	↓	→	PT → aPTT ↑	→	→	–	+	–
TMA (aHUS/TTP)	Complement system/ADAMTS13	Microthrombosis/arterial/venous thrombosis	↓	→〜↑	→	→	→	aHUS +/–TTP –/+	–	–

*DIC* disseminated intravascular coagulation, *SIC* sepsis-induced coagulopathy, *HPS* hemophagocytic syndrome, *APS* antiphospholipid syndrome, *TMA* thrombotic microangiopathy, *aHUS* atypical hemolytic uremic syndrome, *TTP* thrombotic thrombocytopenic purpura, *PT* prothrombin time, *aPTT* activated partial thromboplastin time, *VWF* von Willebrand factor, *IL* interleukin

## Sepsis-induced coagulopathy (SIC) and disseminated intravascular coagulation (DIC)

**Fig. 1:**
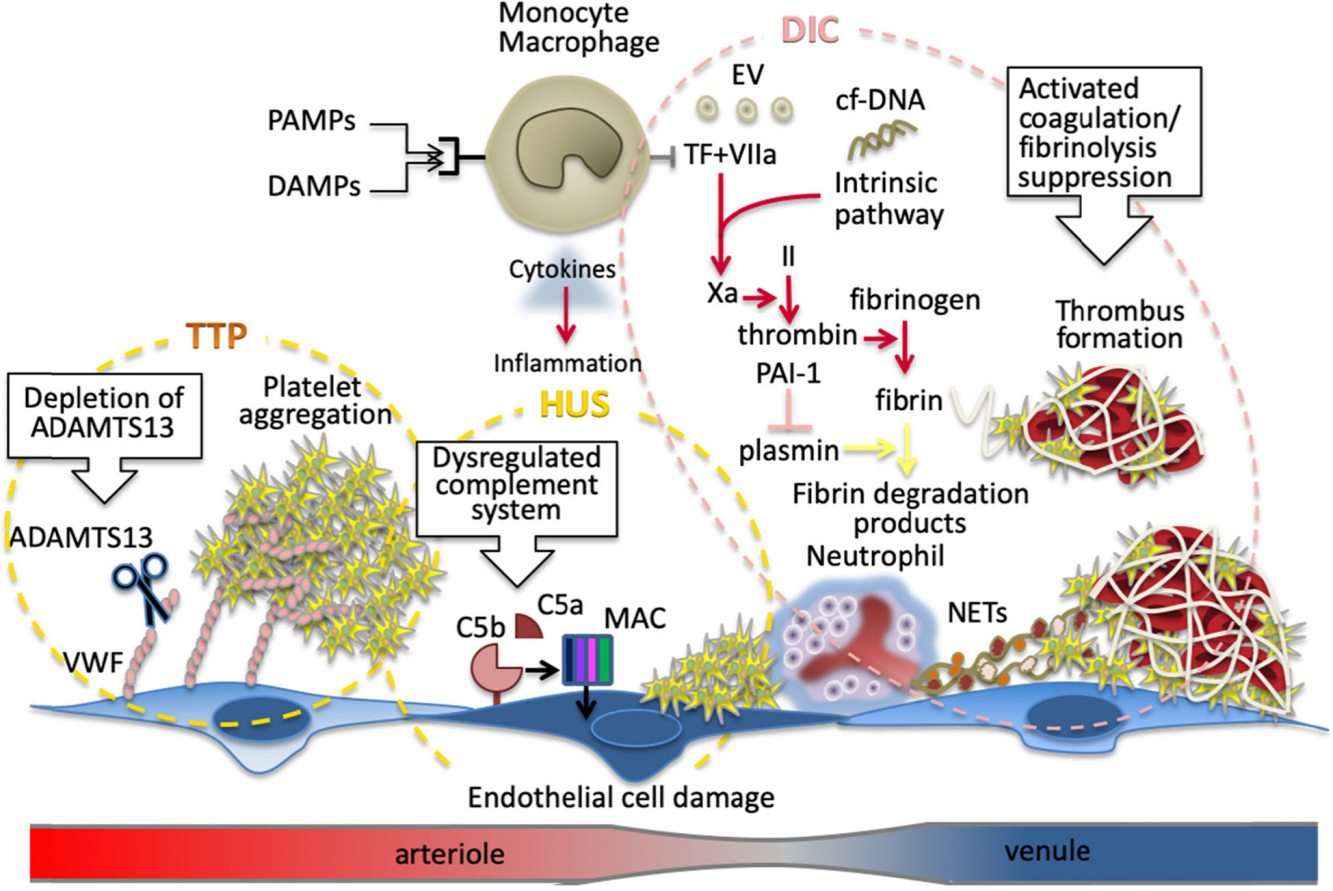
Thrombus formation in disseminated intravascular coagulation, thrombotic thrombocytopenic purpura, and hemolytic uremic syndrome. In bacterial sepsis, immune cells such as monocyte and macrophages are activated by pathogen-associated molecular patterns (PAMPs) and host-derived damage-associated molecular patterns (DAMPs). The immune cells initiate coagulation cascades through expressing tissue factor (TF) and releasing extracellular vesicles (EVs). The activated neutrophils and neutrophil extracellular traps (NETs) are also involved in coagulation. Degradation of fibrin, the end product of coagulation activation, is suppressed by increased levels of plasminogen activator inhibitor-1 (PAI-1). In thrombotic thrombocytopenic purpura (TTP), increased high multimers of von Willebrand factor (VWF) caused by ant-ADAMTS13 antibodies stimulate platelet aggregation. In hemolytic uremic syndrome (HUS), dysregulated complement system and its terminal product, membrane attack protein (MAC), damage vascular endothelial cells, and initiate clot formation

The pathophysiology of bacterial SIC and disseminated intravascular coagulation (DIC) has been extensively studied. Since “inflammation” and “coagulation” are the common keywords in SIC/DIC and CAC, it is helpful to consider prior studies regarding SIC/DIC. The mechanism of procoagulant responses in bacterial sepsis is complex, and various factors, including pathogen-associated molecular patterns (PAMPs) and host-derived damage-associated molecular patterns (DAMPs), are known to trigger the proinflammatory responses and activate systemic coagulation (Fig. 1). Since inflammation and coagulation are both essential host defense mechanisms, the responses increase in proportion to disease severity and can potentially injure the host [10]. Host defense mechanisms include proinflammatory cytokines such as interleukin (IL)-1β, IL-6, tumor necrosis factor-α (TNFα), and complement system proteins, all of which can induce coagulopathy [11]. In addition, tissue factor expression on monocytes/macrophages, neutrophil activation, and neutrophil extracellular traps (NETs) produce activation of thrombosis [12, 13]. This thromboinflammatory response, together with extracellular vesicles, causes endothelial damage that further increase thrombin generation [14, 15]. In SIC/DIC, fibrinolysis is often suppressed due to the over-production of plasminogen activator inhibitor-1 (PAI-1), with progressive fibrin clot formation within the tissue microcirculation leading to organ dysfunction [16]. To detect this type of coagulation disorder, a decrease in the platelet count and increase in prothrombin time (PT)—the two laboratory parameters used in the SIC score—are the most useful indicators [17]. There is a lack of increase in D-dimer levels with increasing SIC/DIC severity due to suppression of fibrinolysis, also called fibrinolytic shutdown [18]. In COVID-19, the D-dimer level is commonly high and usually greater than five times the upper limit of the normal range. Also, in SIC/DIC, anticoagulant proteins such as antithrombin decrease significantly because of increased vascular permeability and other mechanisms [16].

In the case of CAC, other coagulation biomarker changes are relatively minor and abnormalities seen less frequently [2]. Guan et al. [3] reported on over 1000 patients and found a median platelet count of 168 × 10^9^/L in all patients, but only 137.5 × 10^9^/L (median) in the subgroup of patients with severe respiratory disease (all data representing values obtained at hospital admission). They also reported that abnormal D-dimer levels were observed on admission in slightly less than half of the patients. Another report from China also noted that admission platelet counts were lower in non-survivors versus survivors (median values, 122 vs 178 × 10^9^/L, respectively). The median D-dimer value was 2.03 μg/mL in all cases, but even though it was 4.39 μg/mL in non-survivors, the PT was relatively normal (12.6 s) even in the non-survivors [19]. As a result, the incidence of DIC is low in COVID-19 and less than 1% even in severe cases [3, 20]. In another study, Tang et al. [4] reported that 16 out of 183 cases (8.7%) met the DIC criteria of the International Society on Thrombosis and Haemostasis (ISTH), incidences lower than in sepsis where DIC occurs in approximately 30% of cases [4]; moreover, the possibility of superimposed bacterial sepsis, rather than progressive CIVID-19 per se, for progression to DIC cannot be excluded.

Consumptive coagulopathy is a typical feature in SIC/DIC; however, that type of coagulopathy is usually not seen in COVID-19 in its early phase. IL-1β and IL-6 are known to induce thrombocytosis and hyperfibrinogenemia, and sustained inflammation may stimulate the production of these factors [21]. In addition, inflammation and coagulation are localized within the lung in early stages but with disease progression, hypercoagulability becomes systemic and proceeds towards SIC/DIC. The mismatched D-dimer elevation is explained by the upregulation of local fibrinolysis in alveoli by urokinase-type plasminogen activator (u-PA) released from alveolar macrophages [22]. In addition, the direct infection of endothelial cells by the virus (a mechanism that is rather specific for coronaviruses via their cell entry through ACE2 (angiotensin-converting enzyme 2, the receptor for SARS-CoV-2), abundantly expressed on endothelium) leads to a massive release of plasminogen activators [23].

**Fig. 2:**
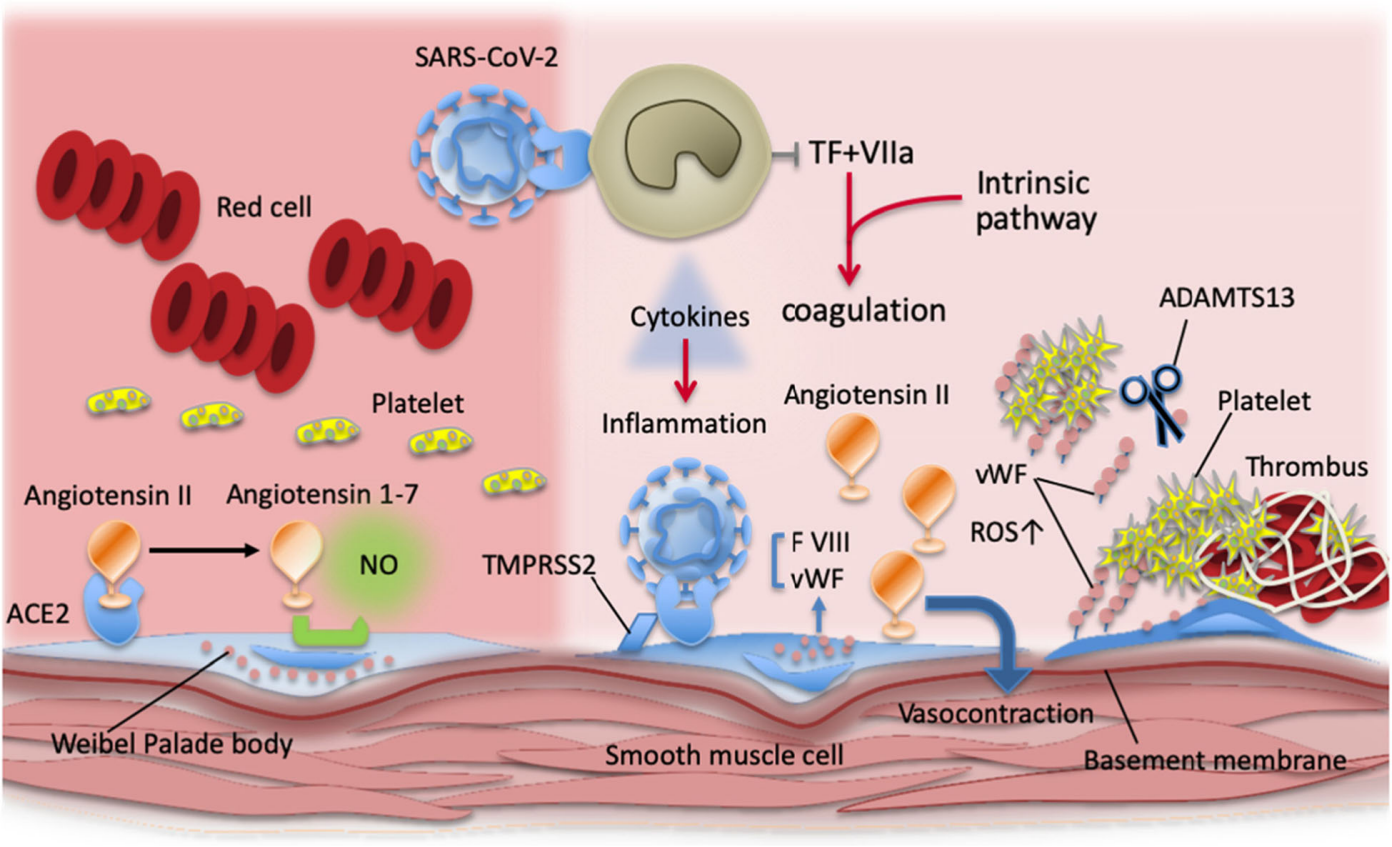
Thrombus formation in COVID-19. In a healthy condition, angiotensin-converting enzyme 2 (ACE2) converts angiotensin II to angiotensin 1–7 which stimulates endothelial cells to produce nitric oxide (NO). NO helps the vessels to vasodilate and suppresses platelet aggregation. In COVID-19, SARS-CoV-2 occupies ACE2 and the angiotensin II level increases, which result in vasoconstriction and decreased blood flow. Von Willebrand factor (VWF) stored in Weibel Palade body is released into the circulation, promoting clot formation. Decreased ADAMTS13 levels (not reported in COVID-19) could contribute to thrombus formation within the vasculature

With an increase in disease severity, there is a procoagulant shift with the acceleration of fibrin formation produced by increased fibrinogen levels and activated platelets. The suppressed fibrinolysis by PAI-1 release accelerates clot formation in the lung capillaries. While ACE2 helps to mediate anticoagulant properties of the vascular endothelium in the healthy state, binding of SARS-CoV-2 to ACE2 aggravates cell damage, upregulates tissue factor expression, and downregulates the protein C system [24] (Fig. 2). In this situation, with or without secondary complications such as tissue hypoxia and concomitant infection, coagulopathy and thrombotic events readily occur.

## Hemophagocytic syndrome (HPS)/hemophagocytic lymphohistiocytosis (HLH)

Hemophagocytic syndrome (HPS) or hemophagocytic lymphohistiocytosis (HLH) is a hyperinflammatory syndrome characterized by the excessive activation of immune cells such as macrophages, natural killer cells, and cytotoxic T cells. Acquired HPS/HLH is due to large amounts of proinflammatory cytokines (TNFα, interferon-γ, IL-1, IL-2, and IL-6) released from activated macrophages and lymphocytes secondary to various triggers including viral infection [25]. The diagnosis is based on five criteria (fever, splenomegaly, decreased counts in two cell lines, hypertriglyceridemia and/or hypofibrinogenemia, and hemophagocytosis) [26]. Recently, three additional criteria were introduced that include low/absent natural killer cell-activity, hyperferritinemia, and high soluble interleukin-2 receptor levels. Although there are some similarities between HPS/HLH and CAC such as the development of “cytokine storm” in COVID-19, the clinical and laboratory findings of the typical HPS/HLH are not common in COVID-19 except fever and hyperferritinemia, with ferritin levels in COVID-19 not usually reaching the extreme high levels often seen in HPS/HLH [27, 28]. A recent retrospective, multicenter study of COVID-19 patients reported elevated ferritin levels in non-survivors versus survivors (1297.6 ng/mL vs 614.0 ng/mL, *P* < 0.01) as well as for IL-6 (11.4 ng/mL vs 6.8 ng/mL, *P* < 0.0001) [29]. Treatment of HPS/HLH requires addressing the causal infection plus immunosuppressive treatments with corticosteroids and/or anticancer chemotherapy for refractory disease [30]. In COVID-19, hemophagocytosis on bone marrow biopsy has not been reported [31]; the use of chemotherapy is not recommended. In contrast to HPS/HLH, severe lung injury and coagulopathy are the dominant characteristics of COVID-19. Direct SARS-CoV-2 infection in the lung epithelial cells followed by the damage to the lung capillary endothelial cells, and subsequent fibrin deposition with upregulated fibrinolysis by u-PA in the alveoli, may contribute to differences between COVID-19 and HPS/HLH [32]. Based on the hypercytokinemia theory, anti-cytokine therapy may have an important role for COVID-19 [33]. However, corticosteroids as are used for HPS/HLH did not improve outcomes in severe acute respiratory syndrome (SARS) and Middle East respiratory syndrome (MERS) patients and resulted in delayed viral clearance [34]. Although research is ongoing, there is no strong evidence at present to support the use of corticosteroids to treat COVID-19.

## Antiphospholipid syndrome (APS)

Thrombotic stroke, reported even in young patients, is a serious complication in COVID-19, with the clinical significance of the presence of antiphospholipid antibodies unknown [35, 36]. Secondary antiphospholipid syndrome (APS) is an acquired autoimmune thrombophilia defined by the development of arterial and venous thromboses in the presence of antiphospholipid antibodies [37]. Antiphospholipid antibodies, i.e., lupus anticoagulant, anticardiolipin, and anti-β_2_-glycoprotein (GP) I antibodies, induce thrombocytopenia and a prolonged activated partial thromboplastin time (aPTT), and these findings often are the clues to APS. Though lung injury is not common in APS, catastrophic antiphospholipid syndrome (CAPS), a rare but highly fatal variant, can result in multiple organ dysfunction, including acute lung injury [38], and the involvement of an over-activated complement system is suspected [39]. Whereas the treatment strategy for the preventing thrombosis in APS can include combined antiplatelet and anticoagulant therapy [40], the benefit of a similar approach of adding antiplatelets to the therapeutic-dose of unfractionated heparin or low molecular weight heparin (LMWH) in COVID-19 patients is unknown and could increase the potential for risk for bleeding; randomized controlled trials are addressing the question in COVID-19 [41]. In addition to anticoagulant therapy, glucocorticoids, and plasma exchange and/or intravenous immunoglobulin, are used to treat CAPS. Convalescent plasma therapy is being developed for COVID-19 [42], but the use of intravenous immunoglobulins has not been studied.

Escher et al. [43] reported an interesting COVID-19 case admitted to the hospital with altered mental status, followed by respiratory and renal failure. The patient demonstrated elevated anticardiolipin and anti-β2-GP I IgM antibodies concurrent with strikingly elevated levels of von Willebrand factor (VWF) and factor VIII. The patient was initially treated with prophylactic LMWH, but with progressive abnormalities in coagulation markers, anticoagulation was switched to therapeutic-dose unfractionated heparin, with clinical improvement. Although the prognostic and treatment implications of APS antibodies and greatly elevated VWF in COVID-19 remain unknown and IgM antibodies are usually not pathogenetic in APS, the authors argued that such unusual laboratory profile suggests a possible role for therapeutic-dose anticoagulation.

## Thrombotic microangiopathy (TMA)

Thrombotic microangiopathy (TMA) is the clinical entity encompassing thrombotic thrombocytopenic purpura (TTP), hemolytic uremic syndrome (HUS), and secondary TMAs. TMA is characterized by thrombus formation in the microvasculature (mainly arterioles) with laboratory findings of microangiopathic hemolytic anemia (MAHA) and thrombocytopenia [44]. The diffuse microvascular thrombi in multiple organs in autopsy cases of COVID-19 are similar to that of TMA, and the changes of hematologic markers resemble those in mild MAHA represented by decreased hemoglobin, increased lactate dehydrogenase (LDH), increased bilirubin, decreased haptoglobin, and appearance of schistocytosis [45].

### Thrombotic thrombocytopenic purpura (TTP)

TTP is caused by autoantibody-induced depletion or inhibition of a disintegrin and metalloproteinase with a thrombospondin type 1 motif, member 13 (ADAMTS13), a metalloprotease enzyme that cleaves large multimers of VWF. In TTP, platelet/VWF microthrombi are found along with severe thrombocytopenia and MAHA. Although acquired TTP can be triggered by infection, to date, depletion of ADAMTS13 in COVID-19 has not been reported. Rather, increased VWF levels in COVID-19 have been reported. Helms et al. [20] found markedly elevated levels of VWF activity, VWF antigen, and factor VIII level in COVID-19. Further, nearly 90% of investigated patients were positive for lupus anticoagulant, suggesting that COVID-19 shows features resembling those of TTP and APS. It is hypothesized that increased VWF is the result of vascular injury since VWF and factor VIII are stored in Weibel-Palade body in endothelial cells. SARS-CoV-2 infection of the endothelial cells may stimulate the release of these components, with levels increasing independently from ADAMTS13 levels. Dengue virus, an RNA virus similar to coronavirus, is known to stimulate endothelial cells to release VWF [46], and an association between elevated circulating levels of VWF and stroke has been reported in dengue [47]. TTP features of thrombocytopenia, fever, decreased consciousness, and renal impairment, all of which can also be seen in COVID-19 [48], suggest possible overlapping pathophysiology can exist. However, arterial thromboembolism, such as stroke and acute coronary syndrome, and microvascular (arteriolar) thrombosis predominate in TTP, whereas venous thromboembolism predominates, with MAHA not commonly seen in COVID-19.

### Hemolytic uremic syndrome (HUS)

HUS can also be induced secondary to infection and results from the dysregulation of the complement pathway. The typical symptoms of HUS are MAHA, acute kidney injury, and other organ dysfunctions [49]. Gavriilaki et al. [50] claim that COVID-19 resembles more the pathophysiology and phenotype of HUS rather than SIC/DIC. The activated complement activates platelets, induces hemolysis, and finally forms the membrane attack complexes (MAC [C5b-9]) that damage the cellular membranes. Though studies of the complement system in COVID-19 are sparse, MERS-CoV is known to increase the levels of C5a and C5b-9 in the blood and lung tissues in a murine model [51]. Furthermore, Margo et al. [52] delineated the deposition of MAC, C4d, and mannose-binding lectin-associated serine protease (MASP) 2 in the lung microvasculature of COVID-19 patients. They also reported that these findings were consistent with sustained, systemic activation of the alternative and lectin-based complement pathways. Complement system activation may be involved in the endothelial damage in COVID-19, and the effect of anticomplement therapy is currently studied [45].

## Heparin-induced thrombocytopenia (HIT)

Heparin-induced thrombocytopenia (HIT) is a prothrombotic complication that can occur following treatment with heparin. As VTE prevention using heparins (unfractionated or LMWH) is emerging as the standard care in COVID-19, patients may be at increased risk of developing HIT. This adverse drug reaction is caused by platelet-activating antibodies that recognize multimolecular complexes of platelet factor 4 (PF4) and heparin. Patients frequently experience moderate-to-severe thrombocytopenia manifesting as venous or arterial thrombi (sometimes both simultaneously). Rarely, a syndrome resembling HIT on both clinical and laboratory grounds—“spontaneous HIT syndrome”—occurs following infection in the absence of heparin therapy [53], but this has not been reported in COVID-19.

The risk of HIT is tenfold lower for LMWH compared with unfractionated heparin, and thus, LMWH is preferred for thromboprophylaxis in COVID-19 [5, 54]. The 4Ts scoring system, consisting of thrombocytopenia, timing of onset, thrombosis, and other causes of thrombocytopenia, is helpful for clinical diagnosis [55], but the application could be challenging in patients with COVID-19. Higher baseline platelet counts in COVID-19 could mask clinical appreciation on HIT-related platelet count declines, so clinical vigilance including appropriate laboratory evaluation for HIT antibodies is needed. When HIT is strongly suspected, anticoagulation should be changed, with options including fondaparinux or direct thrombin inhibitors (e.g., argatroban, bivalirudin) [56].

## Conclusion

**Fig. 3:**
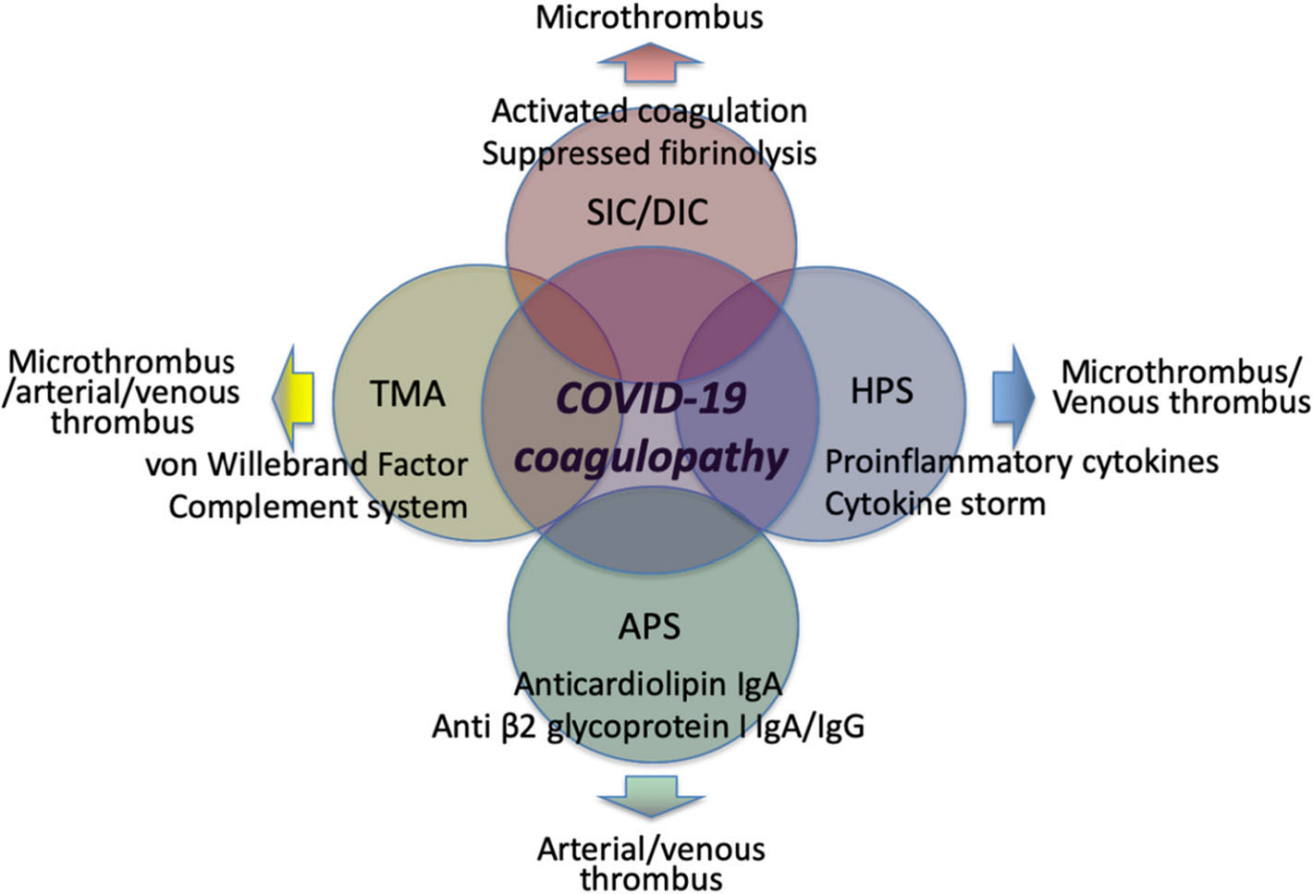
Characteristic features of COVID-19-associated coagulopathy. The clinical and laboratory features of COVID-19-associated coagulopathy (CAC) partially overlap with sepsis-induced coagulopathy (SIC)/disseminated intravascular coagulation (DIC), hemophagocytic syndrome (HPS)/hemophagocytic lymphohistiocytosis (HLH), antiphospholipid syndrome (APS), and thrombotic microangiopathy (TMA); however, it does not perfectly match with any of these other coagulopathies

The number of out-of-hospital sudden death episodes has increased since COVID-19 outbreaks. One of the possible reasons is the high incidence of major thrombotic events in patients with COVID-19; however, the pathogenesis of these life-threatening events is multifactorial and continues to be determined. CAC resembles SIC/DIC, HPS/HLH, APS, and TTP/HUS in some aspects but has unique features that may be defined as a new category of coagulopathy (Fig. 3). Since multiple factors are involved in the development of CAC, further understanding of the underlying pathophysiology is necessary for appropriate management.

## Data Availability

Not applicable.

## Abbreviations

CAC: COVID-19-associated coagulopathy
SIC: Sepsis-induced coagulopathy
DIC: Disseminated intravascular coagulation
VTE: Venous thromboembolism
PAMPs: Pathogen-associated molecular patterns
DAMPs: Damage-associated molecular patterns
IL: Interleukin
TNFα: Tumor necrosis factor-α
NETs: Neutrophil extracellular traps
PAI-1: Plasminogen activator inhibitor-1
PT: Prothrombin time
u-PA: Urokinase-type plasminogen activator
ACE2: Angiotensin-converting enzyme 2
HPS: Hemophagocytic syndrome
HLH: Hemophagocytic lymphohistiocytosis
SARS: Severe acute respiratory syndrome
MERS: Middle East respiratory syndrome
APS: Antiphospholipid syndrome
GP: Glycoprotein
aPTT: Activated partial thromboplastin time
CAPS: Catastrophic antiphospholipid syndrome
LMWH: Low molecular weight heparin
VWF: von Willebrand factor
TMA: Thrombotic microangiopathy
TTP: Thrombotic thrombocytopenic purpura
HUS: Hemolytic uremic syndrome
MAHA: Microangiopathic hemolytic anemia
LDH: Lactate dehydrogenase
ADAMTS13: Metalloproteinase with a thrombospondin type 1 motif, member 13
MAC: Membrane attack complexes
HIT: Heparin-induced thrombocytopenia
PF4: Platelet factor 4

## References

[1] Wu Z, McGoogan JM. Characteristics of and important lessons from the coronavirus disease 2019 (COVID-19) outbreak in China: summary of a report of 72 314 cases from the Chinese Center for Disease Control and Prevention. JAMA. 2020. 10.1001/jama.2020.2648. 10.1001/jama.2020.2648 32091533

[2] Levi M, Thachil J, Iba T, Levy J (2020). Coagulation abnormalities and thrombosis in patients with COVID-19 infection. Lancet Haematol.

[3] Wei L, Liu Y, Hu YH (2020). Clinical characteristics of coronavirus disease 2019 in China. N Engl J Med.

[4] Tang N, Li D, Wang X, Sun Z. Abnormal coagulation parameters are associated with poor prognosis in patients with novel coronavirus pneumonia. J Thromb Haemost. 2020. 10.1111/jth.14768. 10.1111/jth.14768 PMC7166509 32073213

[5] Thachil J, Tang N, Gando S (2020). ISTH interim guidance on recognition and management of coagulopathy in COVID-19. J Thromb Haemost.

[6] Wichmann D, Sperhake JP, Lütgehetmann M, et al. Autopsy findings and venous thromboembolism in patients with COVID-19: a prospective cohort study. Ann Intern Med. 2020. 10.7326/M20-2003. 10.7326/M20-2003 PMC7240772 32374815

[7] Mehra MR, Desai SS, Kuy S, et al. Cardiovascular disease, drug therapy, and mortality in COVID-19. N Engl J Med. 2020. 10.1056/NEJMoa2007621. 10.1056/NEJMoa2007621 PMC7206931 32356626

[8] Tang N, Bai H, Chen X, et al. Anticoagulanttreatment is associated with decreased mortality in severe coronavirus disease 2019 patients with coagulopathy. J Thromb Haemost. 2020. 10.1111/jth.14817. 10.1111/jth.14817 PMC9906401 32220112

[9] Paranjpe I, Fuster V, Lala A, et al. Association of treatment dose anticoagulation with in-hospital survival among hospitalized patients with COVID-19. J Am Coll Cardiol. 2020. 10.1016/j.jacc.2020.05.001. 10.1016/j.jacc.2020.05.001 PMC7202841 32387623

[10] Iba T, Levy JH (2018). Inflammation and thrombosis: roles of neutrophils, platelets and endothelial cells and their interactions in thrombus formation during sepsis. J Thromb Haemost.

[11] Chang JC (2019). Sepsis and septic shock: endothelial molecular pathogenesis associated with vascular microthrombotic disease. Thromb J.

[12] Iba T, Miki T, Hashiguchi N (2014). Is the neutrophil a ‘prima donna’ in the procoagulant process during sepsis?. Crit Care.

[13] Liaw PC, Ito T, Iba T (2016). DAMP and DIC: the role of extracellular DNA and DNA-binding proteins in the pathogenesis of DIC. Blood Rev.

[14] Wang Y, Luo L, Braun OÖ (2018). Neutrophil extracellular trap-microparticle complexes enhance thrombin generation via the intrinsic pathway of coagulation in mice. Sci Rep.

[15] Østerud B, Bjørklid E (2001). The tissue factor pathway in disseminated intravascular coagulation. Semin Thromb Hemost.

[16] Iba T, Levy JH (2020). Sepsis-induced coagulopathy and disseminated intravascular coagulation. Anesthesiology..

[17] Iba T, Levy JH, Warkentin TE (2019). Diagnosis and management of sepsis-induced coagulopathy and disseminated intravascular coagulation. J Thromb Haemost.

[18] Madoiwa S (2015). Recent advances in disseminated intravascular coagulation: endothelial cells and fibrinolysis in sepsis-induced DIC. J Intensive Care.

[19] Wang D, Yin Y, Hu C (2020). Clinical course and outcome of 107 patients infected with the novel coronavirus, SARS-CoV-2, discharged from two hospitals in Wuhan. China Crit Care.

[20] Helms J, Tacquard C, Severac F, et al. High risk of thrombosis in patients with severe SARS-CoV-2 infection: a multicenter prospective cohort study. Intensive Care Med. 2020:1–10. 10.1007/s00134-020-06062-x. 10.1007/s00134-020-06062-x PMC7197634 32367170

[21] Yang M, Ng MH, Li CK (2008). Thrombopoietin levels increased in patients with severe acute respiratory syndrome. Thromb Res.

[22] Gralinski LE, Bankhead A, Jeng S (2013). Mechanisms of severe acute respiratory syndrome coronavirus-induced acute lung injury. mBio.

[23] Varga Z, Flammer AJ, Steiger P (2020). Endothelial cell infection and endotheliitis in COVID-19. Lancet.

[24] Richardson MA, Gupta A, O'Brien LA (2008). Treatment of sepsis-induced acquired protein C deficiency reverses angiotensin-converting enzyme-2 inhibition and decreases pulmonary inflammatory response. J Pharmacol Exp Ther.

[25] Ramachandra S, Zaidi F, Aggarwal A, Gera R (2017). Recent advances in diagnostic and therapeutic guidelines for primary and secondary hemophagocytic lymphohistiocytosis. Blood Cells Mol Dis.

[26] Henter JI, Horne A, Aricó M (2007). HLH-2004: diagnostic and therapeutic guidelines for hemophagocytic lymphohistiocytosis. Pediatr Blood Cancer.

[27] Mehta P, McAuley DF, Brown M (2020). COVID-19: consider cytokine storm syndromes and immunosuppression. Lancet.

[28] Moore JB, June CH (2020). Cytokine release syndrome in severe COVID-19. Science.

[29] Ruan Q, Yang K, Wang W (2020). Clinical predictors of mortality due to COVID-19 based on an analysis of data of 150 patients from Wuhan. China Intensive Care Med.

[30] Kleynberg RL, Schiller GJ (2012). Secondary hemophagocytic lymphohistiocytosis in adults: an update on diagnosis and therapy. Clin Adv Hematol Oncol.

[31] Dimopoulos G, de Mast Q, Markou N, et al. Favorable anakinra responses in severe COVID-19 patients with secondary hemophagocytic lymphohistiocytosis. Cell Host Microbe. 2020. 10.1016/j.chom.2020.05.007. 10.1016/j.chom.2020.05.007 PMC7221383 32411313

[32] Iba T, Levy J, Levi M, et al. Coagulopathy of COVID-19. Crit Care Med 2020 In press. 10.1097/CCM.0000000000004458 PMC7255402 32467443

[33] Radbel J, Narayanan N, Bhatt PJ (2020). Use of tocilizumab for COVID-19-induced cytokine release syndrome: a cautionary case report. Chest.

[34] Channappanavar R, Perlman S (2017). Pathogenic human coronavirus infections: causes and consequences of cytokine storm and immunopathology. Semin Immunopathol.

[35] Zhang Y, Xiao M, Zhang S (2020). Coagulopathy and antiphospholipid antibodies in patients with COVID-19. N Engl J Med.

[36] Bowles L, Platton S, Yartey N, et al. Lupus anticoagulant and abnormal coagulation tests in patients with COVID-19. N Engl J Med. 2020. 10.1056/NEJMc2013656. 10.1056/NEJMc2013656 PMC7217555 32369280

[37] Groot N, de Graeff N, Avcin T (2017). European evidence-based recommendations for diagnosis and treatment of paediatric antiphospholipid syndrome: the SHARE initiative. Ann Rheum Dis.

[38] Wiedermann FJ, Lederer W, Mayr AJ (2003). Prospective observational study of antiphospholipid antibodies in acute lung injury and acute respiratory distress syndrome: comparison with catastrophic antiphospholipid syndrome. Lupus.

[39] Espinosa G, Rodríguez-Pintó I, Cervera R (2017). Catastrophic antiphospholipid syndrome: an update. Panminerva Med.

[40] Garcia D, Erkan D (2018). Diagnosis and management of the antiphospholipid syndrome. N Engl J Med.

[41] Zhou X, Li Y, Yang Q. Antiplatelet therapy following percutaneous coronary intervention in patients complicated by COVID-19: implications from clinical features to pathological findings. Circulation. 2020. 10.1161/CIRCULATIONAHA.120.046988. 10.1161/CIRCULATIONAHA.120.046988 32298134

[42] Shen C, Wang Z, Zhao F (2020). Treatment of 5 critically ill patients with COVID-19 with convalescent plasma. JAMA.

[43] Escher R, Breakey N, Lämmle B (2020). Severe COVID-19 infection associated with endothelial activation. Thromb Res.

[44] Wada H, Matsumoto T, Suzuki K (2018). Differences and similarities between disseminated intravascular coagulation and thrombotic microangiopathy. Thromb J.

[45] Campbell CM, Kahwash R. Will complement inhibition be the new target in treating COVID-19 related systemic thrombosis? Circulation. 2020. 10.1161/CIRCULATIONAHA.120.047419. 10.1161/CIRCULATIONAHA.120.047419 32271624

[46] Tadkalkar N, Prasad S, Gangodkar S (2019). Dengue virus NS1 exposure affects von Willebrand factor profile and platelet adhesion properties of cultured vascular endothelial cells. Indian J Hematol Blood Transfus.

[47] Roldán V, Marín F, García-Herola A, Lip GY (2005). Correlation of plasma von Willebrand factor levels, an index of endothelial damage/dysfunction, with two point-based stroke risk stratification scores in atrial fibrillation. Thromb Res.

[48] Scully M, Hunt BJ, Benjamin S (2012). Guidelines on the diagnosis and management of thrombotic thrombocytopenic purpura and other thrombotic microangiopathies. Br J Haematol.

[49] Azoulay E, Knoebl P, Garnacho-Montero J (2017). Expert statements on the standard of care in critically ill adult patients with atypical hemolytic uremic syndrome. Chest.

[50] Gavriilaki E, Brodsky RA. Severe COVID-19 infection and thrombotic microangiopathy: success doesn’t come easily. Br J Haematol. 2020. 10.1111/bjh. 10.1111/bjh.16783 32369610

[51] Jiang Y, Zhao G, Song N (2018). Blockade of the C5a-C5aR axis alleviates lung damage in hDPP4-transgenic mice infected with MERS-CoV. Emerg Microbes Infect.

[52] Magro C, Mulvey JJ, Berlin D, et al. Complement associated microvascular injury and thrombosis in the pathogenesis of severe COVID-19 infection: a report of five cases. Transl Res 2020: S1931-5244(20)30070–0. 10.1016/j.trsl.2020.04.007 PMC7158248 32299776

[53] Moores G, Warkentin TE, Mohammed AM, et al. Spontaneous heparin-induced thrombocytopenia presenting as cerebral venous sinus thrombosis. Neurol Clin Pract. 2020. 10.1212/CPJ.0000000000000805. 10.1212/CPJ.0000000000000805 PMC8723921 34992986

[54] Martel N, Lee J, Wells PS (2005). Risk for heparin-induced thrombocytopenia with unfractionated and low-molecular-weight heparin thromboprophylaxis: a meta-analysis. Blood.

[55] Warkentin TE (2016). Clinical picture of heparin-induced thrombocytopenia (HIT) and its differentiation from non-HIT thrombocytopenia. Thromb Haemost.

[56] Greinacher A, Selleng K, Warkentin TE (2017). Autoimmune heparin-induced thrombocytopenia. J Thromb Haemost.

